# Deficits in the Mimicry of Facial Expressions in Parkinson's Disease

**DOI:** 10.3389/fpsyg.2016.00780

**Published:** 2016-06-07

**Authors:** Steven R. Livingstone, Esztella Vezer, Lucy M. McGarry, Anthony E. Lang, Frank A. Russo

**Affiliations:** ^1^Department of Psychology, Ryerson University Toronto, ON, Canada; ^2^Department of Computer Science and Information Systems, University of Wisconsin-River Falls Wisconsin, WI, USA; ^3^Toronto Rehabilitation Institute Toronto, ON, Canada; ^4^Division of Neurology, Department of Medicine, University of Toronto Toronto, ON, Canada; ^5^Morton and Gloria Shulman Movement Disorder Centre at The Toronto Western Hospital Toronto, ON, Canada

**Keywords:** facial mimicry, Parkinson's disease, facial expression, emotion, facial masking, facial bradykinesia, hypomimia, rhythm

## Abstract

**Background:** Humans spontaneously mimic the facial expressions of others, facilitating social interaction. This mimicking behavior may be impaired in individuals with Parkinson's disease, for whom the loss of facial movements is a clinical feature.

**Objective:** To assess the presence of facial mimicry in patients with Parkinson's disease.

**Method:** Twenty-seven non-depressed patients with idiopathic Parkinson's disease and 28 age-matched controls had their facial muscles recorded with electromyography while they observed presentations of calm, happy, sad, angry, and fearful emotions.

**Results:** Patients exhibited reduced amplitude and delayed onset in the zygomaticus major muscle region (smiling response) following happy presentations (patients *M* = 0.02, 95% confidence interval [CI] −0.15 to 0.18, controls *M* = 0.26, CI 0.14 to 0.37, ANOVA, effect size [ES] = 0.18, *p* < 0.001). Although patients exhibited activation of the corrugator supercilii and medial frontalis (frowning response) following sad and fearful presentations, the frontalis response to sad presentations was attenuated relative to controls (patients *M* = 0.05, CI −0.08 to 0.18, controls *M* = 0.21, CI 0.09 to 0.34, ANOVA, ES = 0.07, *p* = 0.017). The amplitude of patients' zygomaticus activity in response to positive emotions was found to be negatively correlated with response times for ratings of emotional identification, suggesting a motor-behavioral link (*r* = –0.45, *p* = 0.02, two-tailed).

**Conclusions:** Patients showed decreased mimicry overall, mimicking other peoples' frowns to some extent, but presenting with profoundly weakened and delayed smiles. These findings open a new avenue of inquiry into the “masked face” syndrome of PD.

## Introduction

Parkinson's disease (PD) is the second most common neurodegenerative disorder, affecting 1% of people aged over 60 and rising to 4% of the population over 80 (de Lau and Breteler, 2006). The pathology of PD is defined by the degeneration of dopaminergic neurons in the substantia nigra (Olanow et al., 2009). One of the four cardinal symptoms of PD is akinesia, a loss of movement affecting the body and face (Berardelli et al., 2001; Davie, 2008; Jankovic, 2008). Patients with PD often present with a reduction or loss in the formation of spontaneous emotional facial expressions (Buck and Duffy, 1980; Katsikitis and Pilowsky, 1988, 1991; Saku and Ellgring, 1992; Brozgold et al., 1998) and voluntarily-posed facial expressions (Borod et al., 1990; Bowers et al., 2006; Marsili et al., 2014). Patients smile less frequently and less intensely, and also produce more “false smiles,” leading to an impression of being cold and withdrawn (Pentland et al., 1987; Pitcairn et al., 1990). This loss of facial movements is one of the most distinctive clinical features of PD, and has been termed the “masked face” syndrome (Rinn, 1984; Bologna et al., 2013).

When healthy individuals are exposed to emotional facial expressions, even unconsciously, they spontaneously react with brief automatic facial movements that mimic the presented faces (Dimberg et al., 2000). These movements have been interpreted as a form of mimicking behavior, and are referred to as “facial mimicry” (Lundqvist and Dimberg, 1995; Hess and Blairy, 2001). The presence of these movements have been suggested to improve observers' accuracy and response time during emotional identification, and are thought to play an important role in social communication (Chartrand and Bargh, 1999; Niedenthal et al., 2001; Sonnby-Borgström et al., 2003; Niedenthal, 2007; Oberman et al., 2007; Stel and van Knippenberg, 2008; Wood et al., 2016).

Mimicry is typified by distinct facial muscle patterning following the presentation of emotional facial expressions and voices (Lang et al., 1993; Hietanen et al., 1998; Bradley and Lang, 2000; Hess and Fischer, 2013). Positive expressions of emotion, such as happiness, typically elicit strong activity in the zygomaticus major muscle region, indicative of a smiling response (Dimberg, 1982; Lang et al., 1993; Lundqvist and Dimberg, 1995; Hess and Blairy, 2001; Sato and Yoshikawa, 2007). Negative expressions of emotion, such as sadness, anger, and fear, typically elicit activity in the corrugator supercilii region (Lundqvist and Dimberg, 1995; Dimberg and Thunberg, 1998; Dimberg et al., 2000; Hess and Blairy, 2001; Magnée et al., 2007; Sato and Yoshikawa, 2007; Chan et al., 2014), and to a lesser extent, medial frontalis muscle region (Lundqvist, 1995; Moody et al., 2007), suggestive of a frowning response. In this study we examine the presence of facial mimicry using two positive expressions of emotion, happiness and calm, and three negative expressions of emotion, sadness, anger, and fear.

To our knowledge, there has been no investigation of facial mimicry in patients with PD. Three symptoms of movement dysfunction in PD—bradykinesia, akinesia, and hypokinsea—may affect the presentation of facial mimicry movements. Bradykinesia describes slowness of movement; akinesia denotes paucity of voluntary movement; while hypokinesia refers to decreases in movement amplitude (Berardelli et al., 2001; Jankovic, 2008; Ling et al., 2012). Given the presence of akinesia and hypokinesia in PD (Péron et al., 2012), patients may exhibit an attenuation or absence of facial mimicry movements. If movements are present, their muscle response times may be delayed due to bradykinesia. In healthy individuals, experimental manipulations that block facial mimicry lead to increased response times for the identification of emotion (Niedenthal et al., 2001, see also Oberman et al., 2007). Patients with unilateral facial paralysis have also shown differential effects of emotion recognition and response times in a facial mimicry context (Korb et al., 2016). Thus, an attenuated or absent mimicry response in PD patients may also lead to slower response times of emotional identification.

A tendency in facial mimicry research has been to focus on static photographs, showing only the apex of an expression. However, faces in the real world are rarely static, and there is compelling evidence that the presence of dynamic facial information improves observers' performance on emotional and non-emotional tasks (Bassili, 1979; Atkinson et al., 2004; Cunningham and Wallraven, 2009; Krumhuber et al., 2013). Dynamic facial expressions have been shown to elicit stronger facial mimicry responses than static images (Sato and Yoshikawa, 2007). Pairings of congruent facial and vocal expressions—where the emotion expressed through the face is the same as that of the voice—have also been shown to elicit stronger mimicry responses than incongruent pairings (Magnée et al., 2007). This research suggests that dynamic presentations of congruent face and voice expressions will elicit strong facial mimicry responses in observers. In this study we use dynamic, audio-visual presentations of emotional speech and song to elicit facial mimicry in observers (see also Chan et al., 2014). An advantage of using vocal communication is that it pairs emotionally-congruent faces with voices in an ecologically valid context.

A growing body of research has shown that rhythmic auditory stimulation in a therapeutic context may enhance gross motor movement in PD patients (Thaut et al., 1996; Hackney et al., 2007; Ledger et al., 2008; de Bruin et al., 2010). As the internal clock is thought to be impaired in Parkinson's disease (Artieda et al., 1992; O'Boyle et al., 1996), the provision of an external pulse may act as a replacement by which to synchronize mimicry movement. In this study we examine the effect of rhythmic auditory support across three types of vocal stimuli: speech, singing without a metronome, and singing with a metronome pulse. We expected that patients would show the strongest mimicry response to presentations of singing with a metronome pulse, and weakest responses to presentations of speech.

The aim of the present study was to assess the presence of facial mimicry movements in patients with PD. Facial muscle responses were recorded with electromyography following audio-visual presentations of emotion, in non-depressed patients with idiopathic PD and age-matched controls. We hypothesized that patients would exhibit attenuated facial muscle activity relative to controls. Specifically, that patients' would show reduced activity in the zygomaticus major muscle region following positive expressions (happy, calm) relative to controls, and reduced corrugator supercilii and medial frontalis activity following negative expressions (sad, angry, fearful) relative to controls. We also expected that patients would exhibit a delay in facial muscle activation and behavioral responses relative to controls. Finally, we expected that patients would show increased activity in the zygomaticus major to sung positive expressions relative to spoken positive expressions, and increased activity in the corrugator supercilii and medial frontalis regions to sung negative expressions relative to spoken negative expressions.

## Methods

### Participants

**Figure 1:**
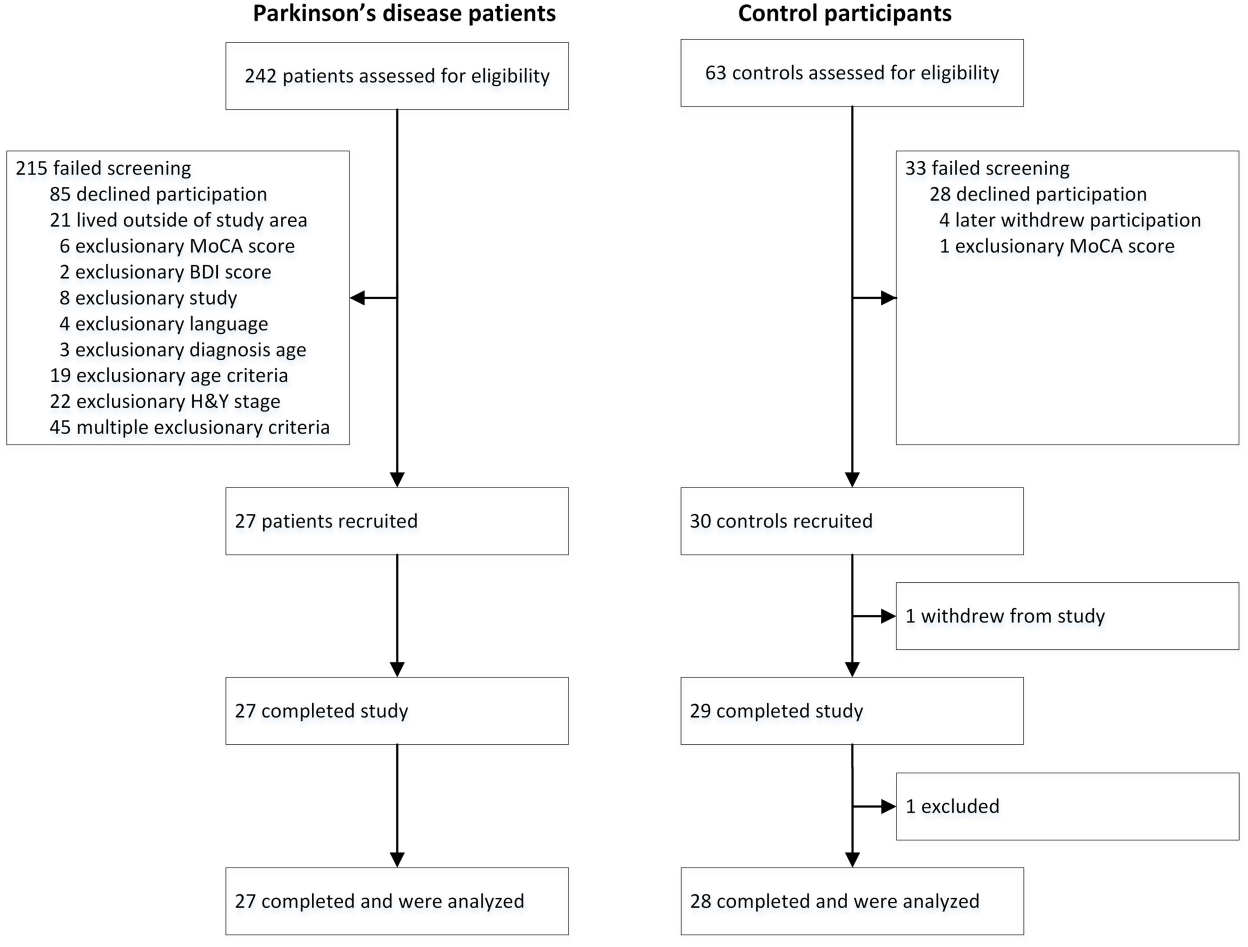
**Flow chart of recruitment, allocation, and participation of study participants**.

The process of recruitment and participation are illustrated in Figure 1. Participant demographics and clinical variables are summarized in Table 1. Twenty-seven patients with idiopathic PD were recruited from the Movement Disorders Clinic at Toronto Western Hospital, and from support groups in the Toronto area. Informed consent was given. Ethical approval for the study was obtained from the University Health Network and Ryerson University, and all patients gave written informed consent. Exclusion criteria included depression (defined as Beck Depression Inventory > 19) (Beck et al., 1961; Schrag et al., 2007), dementia (defined as Montreal Cognitive Assessment < 21) (Nasreddine et al., 2005; Dalrymple-Alford et al., 2010), secondary or acquired parkinsonian disorders (e.g., parkinsonism plus syndromes) (Rao et al., 2003; Brigo et al., 2014), advanced stage PD (defined as Hoehn and Yahr stage > 4) (Goetz et al., 2004), and language unfamiliarity (defined as < 8 years of English speaking experience). General well-being was assessed with the 39-item PD Questionnaire (Peto et al., 1995), and general motor function was assessed with the motor-subscale of the Unified PD Rating Scale (Fahn et al., 1987). Twenty-eight control participants were recruited from the Toronto area through the Ryerson Senior Participant Pool, and were matched according to age and sex.

**Table 1:** **Characteristics of study population^**^a^**^**.

	**Group**		**Test statistics**
	**Parkinson's (*n* = 27)**	**Healthy Control (*n* = 28)**	***t* or χ^2^ (*df*); *p*-value**
Male/Female	18/9	19/9	χ^2^ (1) = 0.01; 0.93
Age, y	68.07 ± 7.54	67.04 ± 7.33	*t*_(53)_ = 0.53; 0.6
Education^b^, y	15.74 ± 2.98	16.18 ± 3.1	*t*_(53)_ = −0.55; 0.7
Years since diagnosis, y	5.59 ± 4.42	N/A	
MoCA	26.11 ± 2.52	26.0 ± 2.47	*t*_(53)_ = −0.56; 0.58
BDI	8.19 ± 5.11	5.0 ± 3.44	*t*_(53)_ = **2.41; 0.02**
Hoehn and Yahr	2.3 ± 0.50	N/A	
PDQ39	28.96 ± 15.56	N/A	
UPDRS-MS^c^	27.07 ± 12.36	N/A	
UPDRS-MS Facial^d^	1.07 ± 0.86	N/A	

*t, Independent-samples t-test; χ^2^, Pearson Chi-Square; MoCA, Montreal Cognitive Assessment; BDI, Beck Depression Inventory; PDQ39, PD Questionnaire; UPDRS-MS, Unified PD Rating Scale—Motor Scale*.

a *Values are mean ± SD. Tests with significant results highlighted in boldface*.

b *Refers to years of formal education starting from the first year of elementary education through to and including tertiary education*.

c *Maximum score of UPDRS-MS was 47, as the pull test was not conducted due to patient safety concerns*.

d *Facial expression sub-score of UPDRS-MS (3.2). Scores range from 0 (normal) to 4 (severe)*.

The sample size of our study was determined using a priori statistical power analysis. We were not aware of any prior study that had examined facial muscle mimicry in PD patients. On the basis of literature concerning facial masking, we predicted a large between-subjects effect size in our mixed design ANOVA *f* = 0.4 (Cohen, 1992). A power analysis using G-Power (Faul et al., 2007) indicated that a total sample size of 44 (22 per group) would be needed, with 95% power (1-β), an alpha of 0.05, and a correlation of 0.5 among the 120 repeated measurements. We were also interested in determining the required sample sizes for our within subjects factors, and between-within interaction tests. For these tests we predicted medium-sized effects in our ANOVA (*f* = 0.25), as the levels of each factor had fewer measurements. The test was coded for two groups, with 95% power (1-β), alpha of 0.05, and a correlation of 0.5 among measures. For the main effect of Emotion (five measurements: calm, happy, sad, angry, fearful), a total sample size of 32 would be needed. For the main effect of vocal Channel (three measurements: speech, song, song-metronome), a total sample size of 44 would be needed. For the interactions Group × Emotion, Group × Channel, and Group × Emotion × Channel, samples sizes of 20, 28, and 12 respectively would be needed. These analyses suggest that our recruited sample of 55 participants (27 Patient, 28 Control) would be sufficient to detect the primary EMG effects of interest.

### Stimuli and apparatus

Stimuli from the Ryerson Audio-Visual Database of Emotional Speech and Song were used (Livingstone and Russo, under review). Recordings consisted of four professional actors (2 male, 2 female) with a high level of acting experience (*M* = 13.25 y, *SD* = 4.71). Two emotionally neutral statements were used (“Kids are talking by the door,” “Dogs are sitting by the door”). Statements were seven syllables in length and were matched in word frequency and familiarity using the MRC psycholinguistic database (Coltheart, 1981). Statements were spoken and sung using five different emotions: calm, happy, sad, angry, and fearful. Two additional emotions, surprise and disgust, were presented in the speech condition only. These emotions were not the focus of the current study and were excluded prior to analysis. An additional set of four trials showing no emotion for each channel was included as a control task. Half of the song trials were overlaid with a metronome that was locked to the timing of each stimulus. Analyzed stimuli consisted of 60 unique recordings, composed of 20 speech utterances, 20 song utterances, and 20 song-metronome utterances. The duration of song utterances (*M* = 4.25 s, *SD* = 0.54 s, range = 4.0 to 6.0 s) differed to that of spoken utterances (*M* = 3.25 s, *SD* = 0.43, range = 3.0–4.0 s), as vocalists were singing to a musical rhythm. Examples of these stimuli are presented in Supplemental Video 1. Perceptual validation data for these stimuli (proportion correct, unbiased hit rates, intensity ratings, response times) are presented in Supplemental Table 1 (taken from Livingstone and Russo, under review).

Stimuli were delivered with Presentation® software (NeuroBehavioral Systems, 2014), on a windows PC connected to a 19-in. flat-screen LCD monitor, at a distance of 0.5 m from the participant. Each trial consisted of five epochs. Trials began with a fixation cross that varied randomly from 4000 to 7000 ms. After the fixation cross, the stimulus was presented to participants. A blank screen was then shown, which varied randomly from 2300 to 3400 ms, to reduce expectancy movements. A forced-choice categorical response screen was then presented, on which participants were required to identify the category of the expressed emotion (Speech options: calm, happy, sad, angry, fearful, surprise, disgust, no emotion. Song options: calm happy, sad, angry fearful, no emotion). After a response was selected, a unidimensional Likert response screen was immediately presented, on which participants rated the intensity of the expressed emotion (1 = not at all intense to 9 = very intense).

Facial muscle activity was recorded with a Biopac MP150 system. Silver-silver chloride (Ag/AgCl) electrodes spaced 25 mm apart were applied over the zygomaticus major and corrugator supercilii muscle regions on the left side of the face, and over the medial frontalis muscle region on the right side of the face (Tassinary et al., 2007). Impedance levels were tested, and electrodes that exceeded 25 kOhm were re-adjusted.

### Design and procedure

The experiment was a 2 × 5 mixed design. Group (two levels: Patient, Control) was the between-subjects factor. Channel (three levels: speech, song, song-metronome) × Emotion (five levels: calm, happy, sad, angry, fearful) × Sex of Actor (2) × Token (2) × Repetition (2) were the within-subjects factors, with 120 trials per participant. Trials were blocked by channel and counterbalanced, with remaining factors presented in random order within each block. The term Channel refers to the type of vocal content produced by the actor in the presented stimulus. The term Token refers to different stimulus recordings that have the same channel-emotion-sex factors. Tokens were used to increase the variability of the stimulus, while controlling the factors of interest. Repetition refers to the presentation of the same stimulus video.

Participants were seated in an immobile chair and told that they would be presented with short videos of people speaking and singing with different emotions. Participants were told that after each video they would be asked to identify the emotion they thought the vocalist was expressing, and to rate the intensity of the expressed emotion. The concepts of emotional category and intensity were explained to participants, and the response scales were shown to participants. Participants used their dominant hand to provide feedback on a numeric keypad. Participants placed their palm on the desktop, with their fingers resting on the keys. The numeric keypad was used to minimize hand movement required for responding. To allow participants to keep their arm comfortably immobile throughout the session, cushions were used to raise the participants' forearms to the height of the desk.

Participants were instructed to respond at a comfortable pace, and not to linger on any one question. While response time tasks typically instruct participants to move as quickly as possible, our instruction was designed to reduce task-related anxiety in patients. Anxiety disorders are comorbid with PD (Shiba et al., 2000), and the presence of anxiety may have affected the EMG recording. PD patients administered their levodopa medication ~1 h prior to testing, and were required to have been in their “on time,” as was verbally confirmed by patients (Pahwa et al., 2006).

### EMG processing

EMG data were bandpass filtered to reduce gross-motor artifacts and high-frequency noise (Butterworth, 6th order, 20 and 400 Hz corner frequencies) (van Boxtel, 2001; De Luca et al., 2010). A notch filter (Butterworth, 4th order, 60 Hz) was used to attenuate AC electrical noise. Data were full-wave rectified and smoothed using a Root Mean Square filter with a 50 ms sliding window and overlap of 49 ms. Time-series data were natural log transformed, and standardized as z-scores within participants and electrode sites to allow for comparisons across muscle sites (Tassinary et al., 2007; Oberman et al., 2009). Data were corrected using a baseline subtraction procedure, where mean EMG activity from −2000 to 0 ms prior to stimulus onset were subtracted from the target window, defined as 0 to +3000 ms after stimulus onset. This procedure produced data where values greater than zero represented an increase in muscle activation relative to the prestimulus baseline. A target window of 3000 ms was selected so that no stimulus was shorter than the target window length.

For muscle latency analyses, data were downsampled to 10 Hz, by averaging values for each 100 ms interval from 0 to +3000 ms, producing a time series of 30 distinct 100 ms intervals per trial. To reduce the number of statistical tests, muscle responses were binned within participant to produce mean EMG responses for positive and negative expressions. For positive expressions, calm and happy trials were averaged to produce a single mean EMG response in the zygomaticus major muscle region. For negative expressions, sad and fearful trials were averaged to produce mean EMG responses, for both the corrugator supercilii and medial frontalis muscle regions. Angry trials were excluded from the latency analysis as these trials did not exhibit a strong mimicry response in the primary EMG analysis. This data reduction process yielded three distinct EMG timeseries for muscle latency analysis: zygomaticus major responses to positive expressions, corrugator supercilii responses to negative expressions, and medial frontalis responses to negative expressions.

### Analyses

We expected specific patterns of muscle activation in response to different emotional presentations. We predicted that happy expressions would elicit large contractions of the zygomaticus major muscle region (Dimberg, 1982; Dimberg et al., 2000; Chan et al., 2014). To our knowledge, calm expressions have not been examined in the context of facial mimicry. Calm is a mild positive expression, involving weak activation of the zygomaticus major muscle region (Tottenham et al., 2009). We expected that calm expressions would elicit a small activation of the zygomaticus muscle region. We further predicted that the negative emotions sad, angry, and fearful would elicit activation of the corrugator supercilii and medial frontalis muscle regions (Ekman and Friesen, 1978; Lundqvist, 1995; Wolf et al., 2005; Oberman et al., 2009; Chan et al., 2014).

Two measures were derived from the EMG data. For our primary EMG analysis we calculated the EMG amplitude over specific muscle regions, which we quantified as the mean activity occurring during the target window (0 and +3000 ms after stimulus onset). For our secondary EMG analysis, we calculated the latency of muscle activity onset using a monotonic function, which we defined as the first time interval after which three successive increases in EMG activity occurred.

Behavioral response times were calculated for emotional identification and emotional intensity perceptual measures, defined as the duration of time from feedback screen display to participant input. As patients were expected to show attenuated mimicry responses, we predicted that patients would show an inverse relationship between facial muscle amplitudes and behavioral response times, for specific emotion-muscle pairs. Specifically, that smaller zygomaticus responses for calm and happy trials would exhibit longer response times, and that attenuated corrugator supercilii and medial frontalis responses for sad, angry, and fearful trials would also show longer response times. These hypotheses were examined with separate two-tailed Pearson correlations. Mean response time values across all calm and happy trials for each participant were correlated with mean zygomaticus values for those trials, while mean response times across all sad, angry, and fearful trials for each participant were each correlated with mean EMG corrugator and mean EMG frontalis values for those trials. Participant response measures for emotional identification accuracy and ratings of emotional intensity were also analyzed (see Supplemental Data 1).

One control participant spoke quietly to themselves throughout the EMG recording session, despite instruction, and was excluded from the data prior to analysis. One patient exhibited dyskinesia in the forehead region. As the dyskinesia movements were restricted to the forehead region, and were not present in any other body region, the corrugator supercilii and medial frontalis data obtained from this patient were excluded prior to analysis.

Demographic and questionnaire measures were analyzed with independent samples *t*-tests. EMG activity and response times were analyzed with mixed-design analyses of variance (ANOVA) and paired samples *t*-tests. Behavioral response times were analyzed with mixed-design ANOVA. For all ANOVAs a custom model was specified, wherein incidental factors not implicated in our predictions were excluded (i.e., sex of actor, token, repetition). Sphericity was assessed with Mauchly's test (Mauchly, 1940)^1^. When the test was significant, Greenhouse–Geisser's correction was applied in cases where ε < 0.75, and Huynh-Feldt correction where ε > 0.75 (as proposed by Girden, 1992; Field, 2009). All effect sizes report partial eta-squared values. Means are accompanied by 95% confidence intervals in square brackets. Pairwise comparisons were adjusted using Bonferonni correction. Statistical tests were conducted in MATLAB 2014a and SPSS 22.0.1.

## Results

### Demographic measures

Participant demographics, cognitive function, and depression were assessed, as summarized in Table 1. No significant differences between groups were found in participant demographics or on cognitive function. A significant difference in BDI scores was found, *t*_(53)_ = 2.41, *p* = 0.019, 95% confidence interval [0.43, 4.7], *d* = 0.65. As expected, patients (*M* = 7.81, *SD* = 4.3, range 1–15) scored higher than controls (*M* = 5.25, *SD* = 3.57, range 1–17). Importantly, all participants were below the threshold for moderate depression (BDI < 20), with a majority of patients (25 of 27) and controls (27 of 28) falling within the range of minimal depression (BDI = 0 to 13). UPDRS facial expression sub-scores (*M* = 1.07, *SD* = 0.86) indicated that patients exhibited only minimal facial masking.

### Primary measures: EMG

Separate two-way mixed-design ANOVAs with the between-subjects factor of Group (2), and within-subjects factors of Channel (3 levels: speech, song-metronome, song) and Emotion (5 levels: calm, happy, sad, angry, fearful) were conducted on participants' mean amplitude for zygomaticus major, corrugator supercilii, and medial frontalis muscle regions, as summarized in Table 2. Significant main effects of Emotion were found on all three muscles, confirming that different presentations of emotion produced distinct patterns of muscle activation that were characteristic of facial mimicry.

**Table 2:** **Summary of Analyses of Variance (ANOVA) on participants' facial muscle responses for zygomaticus major, corrugator supercilii, and medial frontalis muscle regions^**^a^**^**.

**Facial EMG Muscle Region Response**			
**Source**	**Zygomaticus**	**Corrugator**	**Frontalis**
**GROUP (G)**			
*F; p*-value	**5.07; 0.028**	0.04; 0.85	0.18; 0.68
$\eta_{p}^{2}$	0.09		
*MSE*	5.36		
*df, dfe*	1, 53		
**EMOTION (E)**			
*F*	**27.53;**<**0.001**	**27.16;**<**0.001**	**27.3;**<**0.001**
$\eta_{p}^{2}$	0.34	0.34	0.34
*MSE*	2.3	1.56	1.08
*df, dfe*	2.06, 109.24	2.11, 109.73	2.6, 135
**E** × **G**			
*F*	**11.25;**<**0.001**	2.46; 0.09	**3.74; 0.017**
$\eta_{p}^{2}$	0.18		0.07
*MSE*	2.3		1.08
*df, dfe*	2.06, 109.24		2.6, 135
**CHANNEL (C)**			
*F*	**5.27; 0.007**	0.57; 0.57	1.62; 0.20
$\eta_{p}^{2}$	0.09		
*MSE*	0.42		
*df, dfe*	2, 106		
**E** × **C**			
*F*	1.76; 0.11	**3.08; 0.002**	**4.13;**<**0.001**
$\eta_{p}^{2}$		0.06	0.07
*MSE*		0.25	0.32
*df, dfe*		8, 416	5.84, 303.77

*$\eta_{p}^{2}$, partial eta-squared; MSE, mean square error; df, degrees of freedom; dfe, degrees of freedom error*.

a *Statistically significant F-tests (p < 0.05) are highlighted in boldface. The factors G × C and G × E × C were not significant for any analysis, and were not included in the table*.

#### Zygomaticus major muscle region

**Figure 2:**
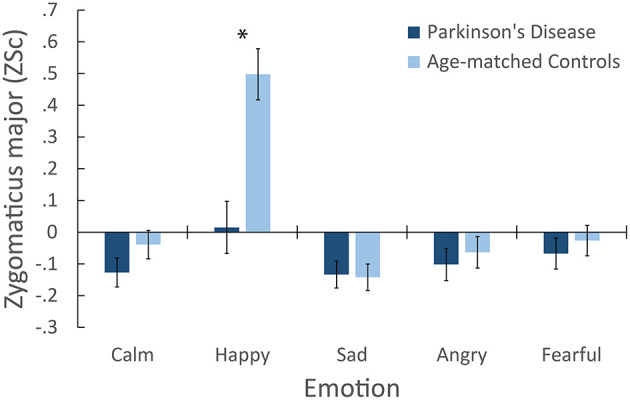
**Mean facial electromyography response for the Zygomaticus major muscle region following emotional presentations, showing the interaction of Group × Emotion**. ^*^Indicates a significant difference in between-group means for that emotion. Error bars denote ± 1 SE.

For zygomaticus major, happy presentations, *M* = 0.26 [0.14, 0.37], elicited larger muscle activity than calm *M* = −0.08 [−0.15, −0.02], sad, *M* = −0.14 [−0.2, −0.08], angry, *M* = −0.08 [−0.15, −0.01], and fearful, *M* = −0.05 [−0.12, 0.02]. A main effect of Group was also found, where controls, *M* = 0.05 [−0.04, 0.13], exhibited more activity overall than patients, *M* = −0.08 [−0.17, −0.001]. Importantly, a significant Emotion × Group interaction was found, as illustrated in Figure 2. *Post-hoc* comparisons (Tukey's HSD = 0.19, α = 0.05) confirmed that responses to happy presentations were larger in controls', *M* = 0.5 [0.34, 0.66], than in patients, *M* = 0.02 [−0.15, 0.18]. Controls' responses to happy presentations were also larger than their responses to other emotions. In contrast, no significant differences were reported between emotions in patients. Overall, these results suggest that controls exhibited characteristic activation of the zygomaticus major in response to happy presentations of emotion. In contrast, patients exhibited little to no differentiation of emotion in the zygomaticus major muscle region.

We further assessed the potential effect of depression on zygomaticus responses. An analysis of covariance (ANCOVA) was conducted on zygomaticus responses, with BDI scores entered as a covariate. BDI was not significant, *F*_(1, 52)_ = 0.35, *p* = 0.56, nor were any interactions involving BDI. The effect of Group was marginally significant, *F*_(1, 52)_ = 3.76, *p* = 0.058, $\eta_{p}^{2}=0.07$. Importantly, the interaction of Emotion × Group remained significant after controlling for BDI scores, *F*_(2.1, 109.15)_ = 7.98, *p* < 0.001, $\eta_{p}^{2}$ = 0.13.

#### Medial frontalis muscle region

**Figure 3:**
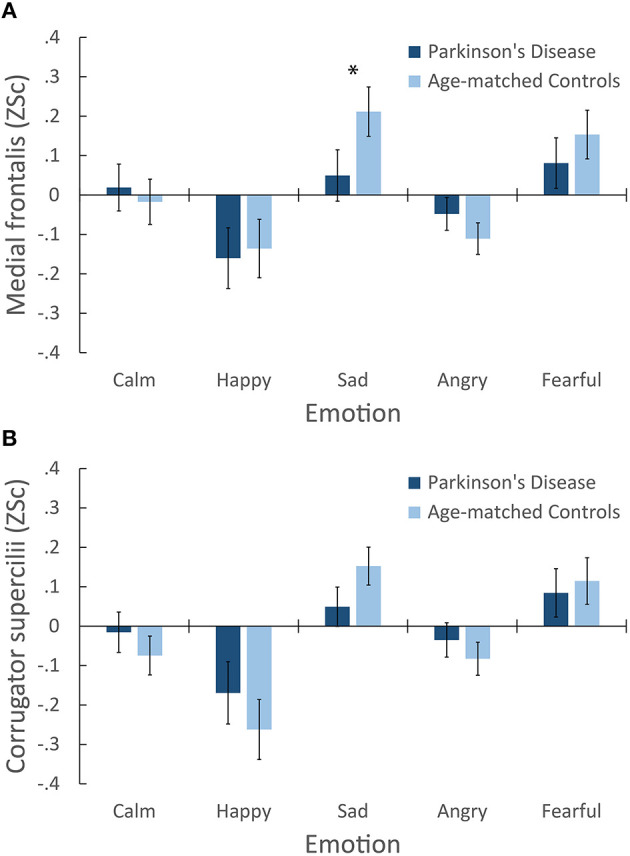
**Mean facial electromyography response following emotional presentations**. Two panes illustrate responses of the **(A)** Medial frontalis region, showing a significant interaction of Group × Emotion. ^*^Indicates a significant difference in between-group means for that emotion. **(B)** Corrugator supercilii muscle region. No interaction of Emotion and Group was found for corrugator responses, however both groups are shown for ease of visual comparison.

For medial frontalis, sad presentations, *M* = 0.13 [0.04, 0.22], and fearful, *M* = 0.12 [0.03, 0.21], elicited larger activation than calm, *M* = 0.001 [−0.08, 0.08], happy, *M* = −0.15 [−0.26, −0.04], and angry, *M* = −0.08 [−0.14, −0.02]. No main effect of Group was found. Importantly, a significant Emotion × Group interaction was found, as illustrated in Figure 3A. *Post-hoc* comparisons (Tukey's, HSD = 0.13, α = 0.05) confirmed that sad presentations elicited larger medial frontalis responses in controls, *M* = 0.21 [0.09, 0.34], than patients, *M* = 0.05 [−0.08, 0.18]. Controls' responses to sad were larger than their responses to calm, *M* = −0.02 [−0.13, 0.1], happy, *M* = −0.14 [−0.29, 0.01], and angry, *M* = −0.11 [−0.19, −0.03], while their responses to fear, *M* = 0.15 [0.03, 0.28] were larger than their responses to calm and happy. Patients' responses to calm, *M* = 0.02 [−0.1, 0.14], sad, and fearful, *M* = 0.08 [−0.05, 0.21] were all larger than their responses to happy, *M* = −0.16 [−0.32, −0.01], while their responses to fearful were also larger than their responses to angry, *M* = −0.05 [−0.13, 0.04]. A significant interaction of Channel × Emotion was also found. *Post-hoc* comparisons (Tukey's HSD = 0.09, α = 0.05) confirmed that responses to calm Song-Metronome, *M* = 0.015 [−0.07, 0.1], were larger than calm Speech, *M* = −0.09 [−0.2, 0.02], while no other emotion varied between channels, suggesting a role in the interaction. Overall, these results suggest that both groups exhibited characteristic activation of the medial frontalis muscle region, with the largest activity occurring in response to the negative emotions sad and fearful, with controls exhibiting a greater response to sad than patients.

#### Corrugator supercilii muscle region

For corrugator supercilii, sad presentations, *M* = 0.10 [0.03, 0.17], and fearful, *M* = 0.10 [0.01, 0.19], elicited larger muscle activity than calm, *M* = −0.05 [−0.12, 0.03], happy, *M* = −0.22 [−0.33, −0.11], and angry, *M* = −0.06 [−0.12, 0.002]. Importantly, no main effect of Group was found, nor were any interactions involving Group. Figure 3B illustrates participants' corrugator supercilii responses. While no interaction of Emotion and Group was found, both groups are plotted to aid comparison with other muscles. A significant interaction of Channel × Emotion was found. *Post-hoc* comparisons (Tukey's HSD = 0.08, α = 0.05) confirmed that responses to calm Song-Metronome, *M* = −0.01 [−0.09, 0.07], were larger than calm Speech, *M* = −0.13 [−0.22, −0.04], while no other emotion varied between channels, suggesting a role in the interaction. Overall, these results suggest that both groups exhibited comparable activation of the corrugator supercilii muscle region, with the largest activation occurring in response to the negative emotions sad and fearful. As expected, these patterns are similar to those obtained for the medial frontalis muscle region, as seen in Figure 3A.

### Secondary measures: behavioral responses and muscle latency

#### Response time measures

A two-way mixed-design ANOVA was conducted on participants' emotion identification response times. A main effect of Channel was found, *F*_(1.73, 91.84)_ = 27.5, *p* < 0.001, $\eta_{p}^{2}=0.34$, where song, *M* = 3130 ms [2746, 3514], had the fastest response times, followed by song-metronome, *M* = 3589 ms [3255, 3924], with speech, *M* = 4170 ms [3749, 4592], exhibiting the slowest response times. A marginally significant main effect of Emotion was also found, *F*_(2.56, 135.41)_ = 2.8, *p* = 0.051, $\eta_{p}^{2}$ = 0.05. Importantly, a significant main effect of Group was found, *F*_(1, 53)_ = 6.35, *p* = 0.015, $\eta_{p}^{2}$ = 0.11, where patients, *M* = 4064 ms [3571, 4556], had slower response times than controls, *M* = 3196 ms [2712, 3680].

A two-way mixed-design ANOVA was conducted on participants' emotional intensity response times. A significant main effect of Channel was found, *F*_(1.47, 78.1)_ = 9.51, *p* = 0.001, $\eta_{p}^{2}$ = 0.15, where song, *M* = 2039 ms [1654, 2423], had faster response times than song-metronome, *M* = 2374 ms [2073, 2675], and speech, *M* = 2410 ms [2004, 2815]. A main effect of Emotion was found, *F*_(3.4, 180.23)_ = 6.38, *p* < 0.001, $\eta_{p}^{2}$ = 0.11. Shortest responses times were found for angry, *M* = 2078 ms [1738, 2418], and fear, *M* = 2149 ms [1849, 2449], then happy, *M* = 2302 ms [1936, 2669], with longest response times for sad, *M* = 2416 ms [2023, 2809], and calm, *M* = 2426 ms [2001, 2850]. Interestingly, no effect of Group was found, *F*_(1, 53)_ = 0.95, *p* = 0.33, with patients, *M* = 2444 ms [1945, 2944], and controls, *M* = 2104 ms [1614, 2595]. These results suggest that while patients were slower than controls at identifying the category of the emotion, they were equivalent to controls in their response times for ratings of emotional intensity.

#### Muscle latency measures

**Figure 4:**
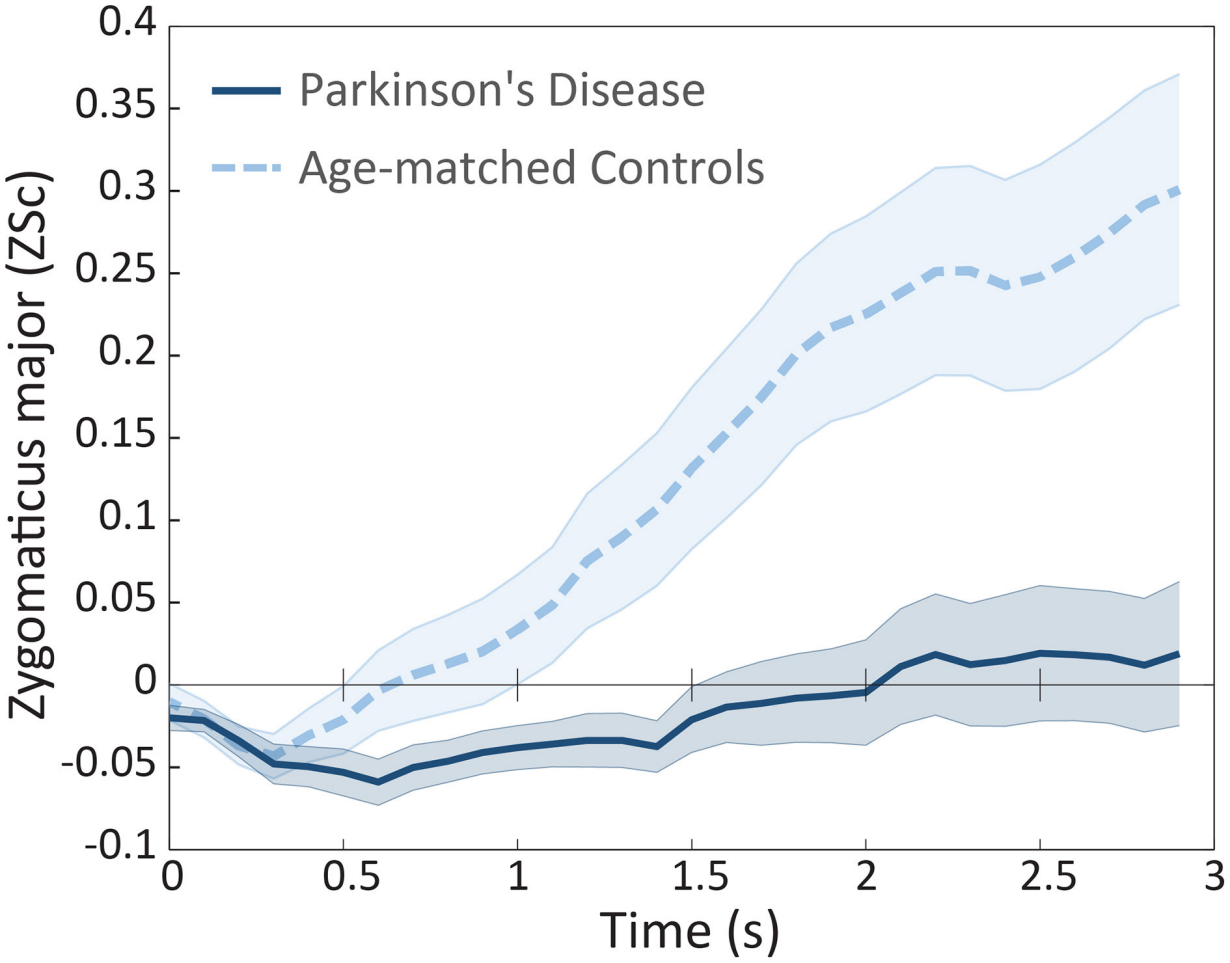
**Mean facial electromyography response for the Zygomaticus major muscle region, in patients and controls, plotted in intervals of 100 ms during the first 3 s of exposure following calm and happy presentations**. Error bars are indicated by shaded regions around trajectory lines, and represent one standard error of the mean.

Next we examined the onset latency of participants' facial muscles following presentations of emotional stimuli. Based on our hypotheses, we examined latency for positive emotions (calm and happy) in the zygomaticus major, and for negative emotions (sad and fearful) in the corrugator supercilii and medial frontalis regions. Zygomaticus muscle onset times in patients (*M* = 1142 ms, *SD* = 719 ms) were significantly delayed relative to controls (*M* = 790 ms, *SD* = 694 ms), *t*_(45)_ = −1.69, *p* < 0.05, *d* = 0.5, as illustrated in Figure 4,^2^. Onset latency was not significantly different for corrugator supercilii (*p* = 0.47) and medial frontalis (*p* = 0.3) muscle regions.

#### Correlating EMG and response time measures

In our final analysis we examined the relationship between patients' emotional identification response times and the amplitude of their facial muscle responses. Patients' mean zygomaticus activity across all positive emotion trials (calm and happy) was correlated with their mean emotion identification response times for those trials. The correlation was statistically significant, *r*_(26)_ = –0.45, *p* = 0.02, two-tailed^3^. Patients mean corrugator supercilii and medial frontalis activity across all negative emotion trials (sad, angry, and fearful) were correlated with their mean emotion identification response times for those trials. The correlation between emotion identification response times and corrugator activity was not significant (*p* = 0.27), nor was the correlation for medial frontalis activity (*p* = 0.16). These results indicate that patients were quicker at identifying positive emotions (calm and happy) when those trials exhibited larger activity in the zygomaticus major muscle region.

## Discussion

Using facial electromyography in the context of an emotion identification task, we provide converging evidence that patients with Parkinson's disease exhibit deficits in facial mimicry. Patients showed little to no reaction in the zygomaticus major muscle region following happy presentations of emotion, while controls exhibited a robust muscle response that was characteristic of facial mimicry (Dimberg, 1982). Zygomaticus responses following happy presentations were also significantly delayed in patients relative to controls, beginning 350 ms later on average. Importantly, these results were unrelated to depression scores (Schwartz et al., 1976). In contrast, both groups exhibited comparable patterns of activity in the corrugator supercilii muscle region, with strong responses to the negative emotions sad and fearful (Lundqvist, 1995; Dimberg et al., 2000). Both groups exhibited differentiable patterns of activation in the medial frontalis region, however patients exhibited a weaker response to sad presentations relative to controls. Collectively, these results suggest that patients exhibited a deficit in their facial mimicry responses to emotional displays, with patients showing weakened and delayed mimicry to smiles but not frowns.

A deficit in the smiling mimicry response may contribute to the observation of individuals with PD as being cold and withdrawn (Pentland et al., 1987; Pitcairn et al., 1990). From infancy, smiling and other facial expressions play a central role in human communication (Tronick et al., 1978). An absence of smiling mimicry may also impact patients' emotional well-being (Davie, 2008), as previous research has found that the frequency, intensity, and duration of zygomaticus major muscle activation predicts self-reported measures of happiness (Ekman et al., 1980; Cacioppo et al., 1986).

A deficit in the zygomaticus muscle region accords with other characterizations of facial movement in PD, where bradykinesia and hypokinesia have been reported in the lower face of patients during smiling (Marsili et al., 2014). Similarly, patients commonly report oro-buccal symptoms—those affecting regions in and around the mouth—including dysphagia, dysarthria, and sialorrhea (Perez-Lloret et al., 2012). These findings support research suggesting that patients exhibit deficits in spontaneous facial expressions (Rinn, 1984; Smith et al., 1996; Simons et al., 2003; Bowers et al., 2006; Assogna et al., 2008).

Our results suggest an overall deficit in mimicry, but with a profoundly weakened and delayed response in the zygomaticus muscle following happy expressions (smiling response). This impairment may be due to the effects of PD on the basal ganglia network and associated motor areas of the brain. The ventral striatal region within the basal ganglia has been linked to the processing and regulation of positive emotions (Hamann and Mao, 2002; Kim and Hamann, 2007), while negative emotions are primarily processed in the medial prefrontal and anterior cingulate cortices (Etkin et al., 2011). Relative differences in responses to happy expressions vs. negative expressions (sad, fearful), may be due in part to the varied state of degeneration in these brain areas.

A facial mimicry deficit in PD patients is likely a consequence of broader motor symptoms including bradykinesia, akinesia, and hypokinsea. However, our results suggest that these motor symptoms have behavioral consequences. Patients were slower at identifying the category of the expressed emotion, taking 868 ms longer on average than controls. One explanation is that patients were slower than controls at providing manual keyboard feedback due to bradykinesia (slowness of movement). However, response times for ratings of emotional intensity were comparable across both groups. Intensity ratings were also provided more quickly than category responses (2274 vs. 3630 ms respectively), suggesting that patients' response times for emotional category do not reflect a “floor” effect of required hand movement time. While these results should be interpreted with caution, they suggest that varying aspects of emotional judgements may be differentially impaired in PD patients.

Behavioral response times were negatively correlated with the amplitude of facial muscle activation in patients, but not controls. In particular, patients were slower at identifying the emotion for calm and happy trials when their zygomaticus responses to those trials were attenuated. A similar relationship has been reported in healthy individuals, where purposeful blocking of facial mimicry leads to increased response times for emotional identification (Niedenthal et al., 2001). Consequently, a motor-behavioral link may exist where impaired mimicry activity in PD patients may contribute to slower response times of emotional processing. However, future studies are needed to properly assess this relationship.

Patients exhibited comparable facial mimicry activity across speech and song presentations. Previous studies have revealed that an auditory rhythm can facilitate motor activities in PD patients. It is unclear why the singing conditions did not facilitate mimicry activity in patients. One explanation is that an external metronome only facilitates periodic motor activities, such as walking, dancing, or tapping. Another explanation is that the benefits of an auditory rhythm are only realizable following a sustained therapeutic intervention (Thaut et al., 1996; Hackney et al., 2007; Ledger et al., 2008; de Bruin et al., 2010).

The present study had several limitations. First, angry stimuli did not elicit characteristic activation of the corrugator supercilii muscle region in healthy participants (Hess and Fischer, 2013). This absense was unexpected, as angry expressions were the second most accurately identified expression by participants (*M* = 79%), and were rated as the most emotionally intense (*M* = 6.62 out of 9) by participants, as described in Supplemental Data 1. A review of angry stimuli indicated that while actors contracted the corrugator supercilii during vocalization, the contractions were relatively brief in duration. The absense of an angry-corrugator response in participants may therefore be due to the lack of sustained corrugator muscle contractions in the angry stimuli. A second limitation of the study is the degree to which participants' facial muscle responses can be interpreted as facial mimicry, rather than emotional mimicry, due to the use of stimuli that presented both facial and vocal expressions of emotion. Facial mimicry, also referred to as the matched motor hypothesis, contends that observers mimic the same facial muscle movements they are presented with (e.g., anger mimics anger). In contrast, emotional mimicry proposes that observers' responses are an interpretation of the presented signal (e.g., anger elicts fear) (Hess and Fischer, 2013). Importantly though, this theoretical distinction does not affect the main results that PD patients show significantly weakened and delayed facial muscle reactions to presentations of emotional faces and voices. Future studies will ideally examine mimicry responses in PD patients to face-only and voice-only expressions. The third limitation of the study was the use of stimuli that varied in duration, and the effect this may have had on participants' behavioral response times. Spoken stimuli were shorter in duration than sung stimuli. This difference may partly explain why participants were slower at identifying emotion in speech.

## Conclusion

When healthy individuals are exposed to emotional facial expressions, even below the threshold of consciousness, they spontaneously react with muscle activations that mimic the presented faces. The presence of these movements facilitates a range of social and emotional tasks. In the present study, we showed that patients with idiopathic PD exhibited deficits in the mimicry of others' expressions. Patients mimicked other people's frowns to some extent, but mimicked their happy faces with faint smiles that were delayed in onset. These outcomes may have implications for the social well-being of patients, and open a new line of inquiry into the “masked face” syndrome of PD.

## Author contributions

SL was involved in all stages of the research project, statistical analyses, and the preparation and review of the manuscript. EV was involved in the execution of the project, review of statistical analyses, and writing and review of the manuscript. LM was involved in the conception and organization of the project, review of the statistical analyses and manuscript. AL was involved in the conception of the project, review of the statistical analyses and manuscript. FR was involved in the conception and organization of the project, review of the statistical analyses, and writing and review of the manuscript.

## Funding

This research was supported by grants from the Parkinson Society of Canada (2012-22 to FR and SL), and the Natural Sciences and Engineering Research Council of Canada (341583-2012 to FR). All authors ensure that no conflict of interest, financial or otherwise, exists that may be seen as having influenced the research.

### Conflict of interest statement

The authors declare that the research was conducted in the absence of any commercial or financial relationships that could be construed as a potential conflict of interest.

## Footnotes

^1^The assumption of sphericity—that variances of the differences between levels of the independent variables are equal—is an important requirement ANOVA, as the presence of sphericity may result in an inflated F-ratio (Hinton et al., 2014).

^2^In the calculation of zygomaticus latency responses, two healthy controls and nine patients did not satisfy the condition for three consecutive increases, and were not included in the calculation.

^3^The analysis was repeated for controls, though no differences were expected due to a priori predictions of healthy mimicry function. No correlation reached significant across the three muscle regions: zygomaticus (*p* = 0.58), corrugator (*p* = 0.81), and medial frontalis (*p* = 0.19).

## Supplementary material

Supplemental Data 1 **Supplemental results for patients' and controls' measures of emotional identification accuracy and ratings of emotional intensity**.

Supplementary Figure 1 **Supplemental figure accompanying Supplemental Data 1**.

Supplementary Table 1 **Supplemental table containing perceptual validation results for stimuli used in the present study (taken from Livingstone and Russo, under review)**. Validation data includes proportion correct scores, unbiased hit rates, intensity ratings, and responses times for these measures.

Supplemental Video 1 **Supplemental movie containing examples of stimuli used in the present study showing calm, happy, sad, angry, and fearful speech**.
