# Supplementary material for: Deficits in the Mimicry of Facial Expressions in Parkinson's Disease
Source: Front Psychol. 2016 Jun 7;7:780. doi: 10.3389/fpsyg.2016.00780 (PMC4894910; doi:10.3389/fpsyg.2016.00780)
Supplement: Supplemental Data 1 — Supplemental results for patients' and controls' measures of emotional identification accuracy and ratings of emotional intensity. [file DataSheet1.DOCX]

Supplementary results

# Behavioral measures: Accuracy and intensity ratings

A two-way mixed-design analysis of variance (ANOVA) with the between-subjects factor of Group (2 levels: patient, control) and within-subjects factors of Channel (3 levels: speech, song-metronome, song) and Emotion (5 levels: calm, happy, sad, angry, fearful) was conducted on participants’ mean accuracy (% correct) measures. A main effect of Channel was found, *F*(1.6, 86.7) = 38.48, *p* < .001, $\text{η}_{\text{p}}^{\text{2}}$ = .42. Pairwise comparisons confirmed that song-metronome, *M* = .79, 95% confidence interval [.76, .82], was identified more accurately than song, *M* = .75, [.72, .78], and speech, *M* = .67, [.64, .71]. Speech was identified least accurately, as was expected, given the larger number of emotion response options in speech (8 emotion options, correctness chance estimate = 0.125) than in song (6 emotion options, correctness chance estimate = 0.167). A main effect of Emotion was found, *F*(3.5, 184.96) = 26.33, *p* < .001, $\text{η}_{\text{p}}^{\text{2}}$ = .38. Pairwise comparisons confirmed that calm, *M* = .66, 95% CI [.61, .71], fearful, *M* = .68, [.64, .73], and sad, *M* = .69, [.64, .73], were least accurately identified, then angry, *M* = .79, [.76, .82], with happy, *M* = .88, [.84, .92] identified most accurately. A significant interaction of Emotion × Group was found, *F*(3.5, 184.96) = 2.58, *p* = .046, $\text{η}_{\text{p}}^{\text{2}}$ = .05, as illustrated in Supplemental Figure 1. Post hoc comparisons (Tukey’s HSD = .086, α = .05) confirmed that patients identified fearful presentations, *M* = .63 [.57, .69], less accurately than controls, *M* = .73 [.67, .79], suggesting a role in the interaction. All other emotions were identified with comparable accuracy by both groups. A significant Channel × Emotion interaction was also found, *F*(7.3, 387.82) = 12.92, *p* < .001, $\text{η}_{\text{p}}^{\text{2}}$ = .20, suggesting hit rate scores varied with the channel and emotional category. Posthoc comparisons (Tukey’s HSD) confirmed that all emotions except calm were identified least accurately in speech, while calm was identified with comparable accuracy in all channels, suggesting a role in the interaction. Collectively, these results suggest that while participants’ accuracy varied depending on the channel and emotion, the two groups were relatively equivalent in their accuracy of emotional identification.

To correct for possible response bias in multi-level response tasks, the analysis was repeated for the data converted to unbiased hit rates ([Wagner 1993](#_ENREF_88)). For ease of readability, pre-transformed unbiased hit rate means (0-1) are reported. The same two-way repeated measures ANOVA was conducted on observers’ unbiased hit rate scores with Vocal Channel (2) and Emotion (3) entered as within-subjects factors. A main effect of Channel was found, *F*(2, 106) = 9.72, *p* < .001, $\text{η}_{\text{p}}^{\text{2}}$ = .16. Pairwise comparisons confirmed that speech, *M* = .57, 95% CI [.53, .61], was identified less accurately than song-metronome, *M* = .65, [.61, .69], and song, *M* = .64, [.60, .68]. This result was expected given the larger number of emotion response options in speech. A main effect of Emotion was found, *F*(4, 212) = 33.06, *p* < .001, $\text{η}_{\text{p}}^{\text{2}}$ = .38. Pairwise comparisons confirmed that calm, *M* = .51, 95% CI [.47, .55], was least accurately identified, then happy, *M* = .60, [.57, .63] and fearful, *M* = .60, [.56, .64], with sad, *M* = .70, [.66, .75], and angry, *M* = .68, [.64, .72] identified most accurately. No effect of Group was found, *F*(1, 53) = .1, *p* = .75. A marginally significant interaction of Emotion × Group was found, *F*(4, 212) = 2.21, *p* = .069, $\text{η}_{\text{p}}^{\text{2}}$ = .04. A significant Channel × Emotion interaction was also found, *F*(6, 316.68) = 29.67, *p* < .001, $\text{η}_{\text{p}}^{\text{2}}$ = .36, suggesting hit rate scores varied with the channel and emotional category. Overall, unbiased hit rate measures replicated the results of the analyses on raw hit rates.

A two-way mixed-design ANOVA was conducted on participants’ emotional intensity ratings. No effect of Channel was found, *p* = .073. A main effect of Emotion was found, *F*(2.12, 112.08) = 39.46, *p* < .001, $\text{η}_{\text{p}}^{\text{2}}$ = .43. Pairwise comparisons confirmed that angry, *M* = 6.62, [6.35, 6.89], was rated most intense, followed by happy, *M* = 6.4 [6.35, 6.89], fearful, *M* = 6.14 [6.35, 6.89], calm, *M* = 5.46 [6.35, 6.89], and sad, *M* = 5.76 [6.35, 6.89]. A significant Channel × Emotion interaction was found, *F*(7, 371.02) = 23.96, *p* < .001, $\text{η}_{\text{p}}^{\text{2}}$ = .31. No main effect or any interaction with Group was found. These results suggest that while participants’ ratings varied with channel and emotion, the two groups rated presentations with comparable levels of emotional intensity.

##
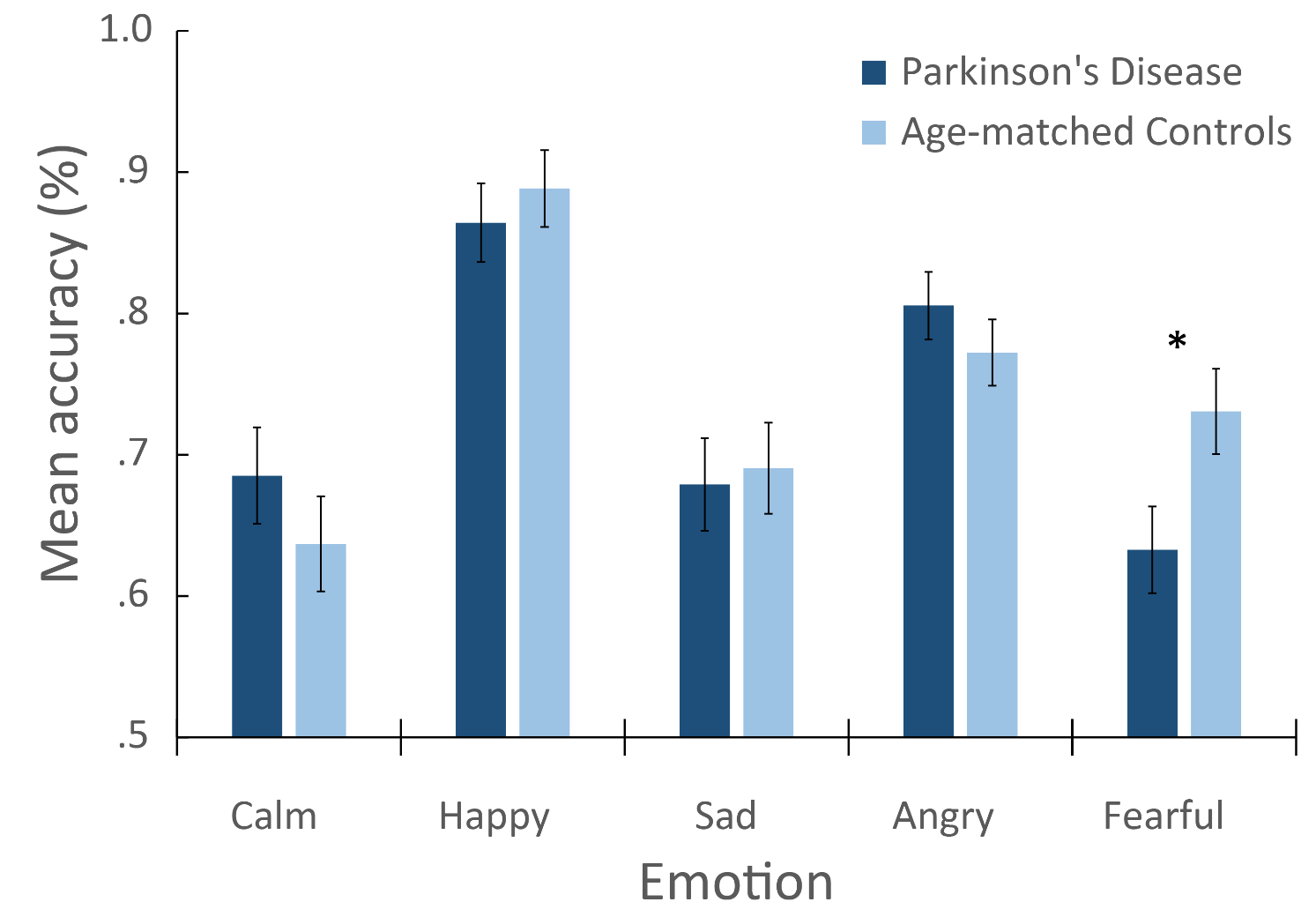


## Supplemental Figure 1: Participants’ mean accuracy (% correct) for the emotional identification task, showing the interaction of Group × Emotion. * indicates a significant difference in between-group means for that emotion. Error bars denote ± 1 SE.
