# Supplementary figures and images for: Deficits in the Mimicry of Facial Expressions in Parkinson's Disease
Source: Front Psychol. 2016 Jun 7;7:780. doi: 10.3389/fpsyg.2016.00780 (PMC4894910; doi:10.3389/fpsyg.2016.00780)

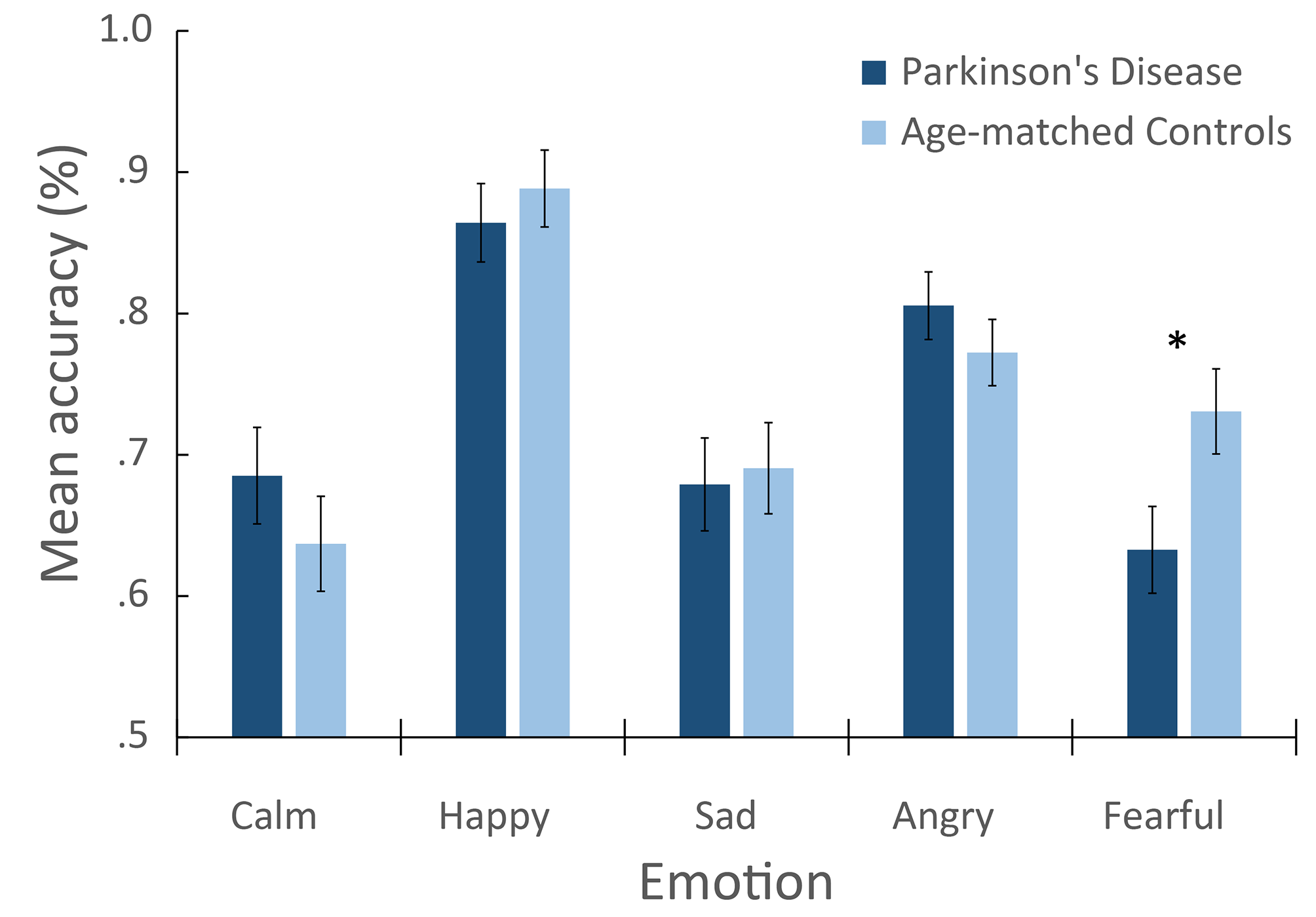

Supplement: Supplementary Figure 1 — Supplemental figure accompanying Supplemental Data 1. [file Image1.TIF]
